# Supplementary material for: Surface Microbiome Profiling of Dental Elevators Using Third‐Generation Sequencing: Implications for Infection Control in Dental Practice
Source: Microbiologyopen. 2025 Dec 8;14(6):e70178. doi: 10.1002/mbo3.70178 (PMC12685768; doi:10.1002/mbo3.70178)
Supplement: Supplementary file 1 — customer_backup. [file MBO3-14-e70178-s001.zip › customer_backup/customer_backup/taxa_summary/krona/groups/treat/M08.Krona.html]

Javascript must be enabled to view this page.

members
magnitude
magnitudeUnassigned

M08.krona

48350

48330

0

0

0

0

0

0

0

0

0

0

0

0

0

0

0

0

0

0

0

0

0

0

0

0

0

0

0

0

0

0

0

0

0

0

0

0

0

0

0

0

0

0

0

4

4

0

0

0

0

0

0

0

0

0

0

0

0

0

0

0

0

0

4

1

1

1

0

0

0

0

0

1

1

1

0

0

1

1

1

0

0

0

0

0

0

0

0

0

0

1

1

1

0

0

0

0

0

0

0

0

0

0

0

0

0

0

0

0

0

0

0

0

0

0

0

0

0

0

57

16

16

0

0

0

16

12

12

0

4

4

0

0

0

0

0

0

0

0

0

0

0

0

0

0

0

0

0

0

0

0

0

0

0

0

0

0

0

0

0

0

0

0

0

0

0

0

0

0

0

0

0

0

0

0

0

0

0

0

1

1

1

0

0

1

1

40

40

0

0

0

0

0

0

0

0

0

0

0

0

0

0

0

0

0

0

0

0

0

0

0

0

40

0

0

0

0

0

40

40

0

0

0

0

0

0

0

0

0

0

0

0

0

0

0

0

0

0

0

0

0

0

0

0

0

0

0

0

0

0

0

0

0

0

0

0

0

0

0

0

0

0

0

0

0

0

0

0

0

0

0

0

0

0

0

0

0

0

0

0

0

0

0

0

0

0

0

0

0

0

0

1

1

1

1

0

0

1

1

0

0

0

0

0

0

0

0

0

0

0

0

0

0

0

0

0

0

0

0

0

0

0

0

0

0

0

0

43

38

38

0

0

0

38

38

38

0

0

0

0

0

0

0

0

0

0

0

0

0

0

0

0

5

0

0

0

0

0

0

0

0

0

0

0

0

0

0

0

0

0

0

0

0

0

0

0

0

5

0

0

0

0

0

0

0

0

5

0

0

5

5

0

0

0

0

0

0

0

0

0

0

0

0

0

0

0

0

0

0

0

0

0

0

0

0

0

0

0

0

0

0

0

0

0

2466

385

0

0

0

0

43

0

0

0

43

0

0

0

0

0

0

0

0

43

0

43

0

0

0

0

54

54

0

0

0

54

54

0

0

1

0

0

0

1

0

0

0

0

0

0

1

1

0

0

0

2

2

1

0

0

1

1

0

0

0

1

0

0

0

0

0

0

0

7

7

1

1

1

1

0

0

0

0

0

0

0

0

0

0

0

0

0

0

0

0

0

0

0

0

0

0

0

0

0

0

0

0

0

0

0

0

0

0

0

0

5

0

0

1

0

0

0

0

0

0

1

0

0

0

0

0

0

0

0

2

0

1

0

0

0

0

0

0

0

0

0

0

0

0

0

0

0

0

0

0

0

0

0

0

0

64

0

0

0

0

0

0

0

0

0

0

0

0

0

0

0

0

0

0

0

0

0

3

0

0

0

3

0

3

0

0

0

0

0

0

0

0

0

0

0

0

0

0

0

0

0

0

0

0

0

0

0

30

0

0

0

0

0

0

0

13

13

0

0

0

0

9

9

0

0

0

0

0

1

1

3

0

0

3

0

0

0

0

0

0

4

3

0

0

0

0

0

0

0

0

0

0

1

0

0

0

0

0

0

0

0

0

0

0

0

0

0

0

0

0

0

0

0

0

0

0

0

0

0

0

0

0

0

0

0

0

0

0

0

0

0

0

0

0

0

0

0

0

0

0

3

0

0

0

1

1

2

2

0

0

0

0

0

0

0

0

0

0

0

0

0

0

0

0

0

0

28

0

0

0

0

0

0

0

0

0

0

0

0

16

0

0

0

0

15

0

0

0

0

0

1

0

0

0

0

0

0

0

0

0

0

0

0

0

0

0

0

12

12

0

0

0

0

0

0

91

0

0

0

0

0

91

0

0

0

0

0

0

0

0

0

0

0

0

0

0

0

91

91

0

0

0

0

0

0

0

0

0

0

0

0

0

0

0

0

0

0

0

0

0

0

0

0

0

0

0

0

0

0

0

0

0

0

0

0

0

0

0

0

0

0

0

0

0

0

0

0

0

0

0

0

0

118

118

1

1

0

0

0

38

38

79

78

0

0

0

0

0

1

0

0

0

0

0

0

0

0

0

0

0

0

0

0

0

0

0

0

0

0

0

0

0

0

0

0

0

0

0

0

0

0

0

0

0

0

0

0

0

0

0

0

0

0

0

0

0

0

0

5

5

0

0

0

0

0

0

0

0

0

0

0

0

0

0

0

0

0

0

0

0

0

0

0

2

2

1

0

1

0

0

0

0

0

0

0

0

0

0

2

0

0

2

0

0

0

0

0

0

0

0

2081

0

0

0

0

0

0

0

0

0

0

0

0

0

0

0

0

0

0

0

1019

61

0

0

61

61

0

0

0

0

0

0

0

4

0

0

0

0

0

0

0

0

0

4

4

0

0

0

0

0

0

0

0

0

0

0

407

0

0

0

0

320

320

87

0

1

85

1

0

0

0

0

0

0

0

0

0

0

0

0

0

0

0

0

0

3

0

0

0

0

2

0

1

1

1

1

0

0

0

0

0

0

0

0

54

0

0

5

5

0

0

0

0

0

0

42

0

42

0

7

7

21

16

16

0

0

3

0

3

2

0

2

0

0

0

0

469

0

0

0

0

0

0

0

0

0

0

0

0

0

0

0

0

0

0

0

0

0

205

0

205

0

0

1

0

1

84

0

84

0

0

0

19

7

12

160

160

0

0

0

0

0

0

0

0

1

1

0

0

0

1

1

0

0

0

0

0

0

0

0

0

0

0

0

0

0

0

0

0

0

0

0

0

135

135

135

81

54

0

0

0

0

0

0

0

0

0

0

0

0

0

0

0

0

0

0

0

34

0

0

0

18

9

8

1

0

0

0

0

8

8

1

0

0

0

1

0

0

0

0

0

0

0

0

0

0

0

0

0

0

0

0

0

0

1

1

1

15

15

0

0

14

1

0

0

0

0

0

0

0

0

0

0

0

0

0

0

0

0

0

0

0

0

0

0

0

0

0

0

0

0

0

0

0

0

0

0

0

0

0

0

0

0

0

0

0

0

0

0

0

0

0

0

0

0

0

0

0

0

0

0

692

4

2

2

2

2

2

2

2

3

0

0

0

3

3

0

0

0

619

579

579

4

4

36

36

0

0

2

2

1

1

0

0

0

0

0

0

4

4

4

58

0

0

0

0

4

4

0

0

26

0

2

24

0

28

28

0

0

0

0

0

0

0

0

0

0

0

0

0

0

0

0

0

0

0

0

0

0

0

0

0

0

0

0

0

0

0

0

0

0

186

0

0

0

0

0

0

0

0

0

0

0

0

0

186

186

186

0

0

0

0

0

0

0

0

0

0

0

0

0

0

0

0

0

0

0

0

0

0

0

0

0

0

0

0

0

0

0

0

0

0

0

0

0

0

0

0

0

0

0

0

0

0

0

0

13

13

0

0

0

0

0

0

0

0

0

0

0

0

13

3

0

10

0

0

1

1

1

1

0

0

0

0

0

0

0

0

0

0

0

0

0

0

0

0

0

0

0

0

0

0

0

0

0

0

0

0

0

0

0

0

0

0

0

0

0

0

0

0

0

0

0

0

0

0

0

0

0

0

0

0

0

0

0

0

0

0

0

0

0

0

0

0

0

0

0

0

0

0

0

0

0

0

0

0

0

0

0

0

0

0

0

0

0

0

0

0

0

0

0

0

0

0

0

0

0

0

0

0

0

0

0

0

0

0

0

0

0

0

0

0

0

0

0

0

0

0

0

0

0

0

0

0

0

0

0

0

0

0

0

0

0

0

0

0

0

0

0

0

0

0

0

0

0

0

0

0

0

0

0

0

0

9956

0

0

0

0

0

9956

1

0

0

0

1

0

0

0

0

0

0

0

0

0

0

0

0

0

0

0

0

0

0

0

0

1

0

0

1

0

0

0

0

0

0

0

0

0

0

0

0

0

0

0

0

0

0

0

0

0

0

0

0

0

0

0

0

0

0

0

0

0

0

0

0

0

0

669

0

0

0

664

0

0

0

0

0

0

0

0

0

0

0

0

0

0

0

0

0

0

0

0

0

664

2

50

555

57

0

0

0

0

0

0

0

0

0

0

0

0

0

0

0

0

0

0

0

4

4

0

4

0

0

0

0

0

1

0

0

0

0

1

1

0

0

9176

4

0

0

4

4

8

8

8

10

10

10

77

0

0

0

0

26

26

51

51

40

0

0

0

0

0

33

0

0

0

33

0

1

1

6

0

4

0

0

0

0

0

2

0

0

1078

1078

1078

72

0

0

0

0

72

0

0

72

0

0

0

0

0

0

0

0

72

72

0

0

1

1

2

0

0

0

0

0

3

0

5

1

0

0

59

735

1

1

702

638

64

32

0

0

31

1

0

0

5082

0

0

0

2

0

2

526

41

0

23

461

1

0

0

1

1

0

0

2225

26

434

106

196

8

0

160

12

13

5

80

5

44

44

0

0

1092

2328

0

56

49

255

431

208

0

0

0

0

366

64

28

0

208

663

0

0

0

0

0

0

1740

1740

0

573

0

1135

0

32

0

0

0

258

258

258

110

0

0

0

0

0

0

0

0

0

0

0

0

110

0

0

0

0

0

0

110

22

88

0

0

0

0

0

0

0

0

0

0

0

0

0

0

0

0

0

0

0

0

0

0

0

0

0

0

0

0

0

0

0

0

0

0

0

0

0

0

0

0

0

0

0

0

0

0

0

0

0

0

0

0

0

0

0

0

0

0

0

0

0

0

0

0

0

0

0

0

0

0

0

0

0

0

0

0

0

0

0

0

0

0

0

0

0

0

0

0

0

0

0

0

0

0

0

0

0

0

0

0

0

0

0

0

0

0

0

0

0

0

0

0

0

0

0

0

0

0

0

0

0

0

0

0

0

0

0

0

0

0

0

0

0

0

0

0

0

0

0

0

0

0

0

0

0

0

0

0

0

0

0

0

0

0

0

0

0

0

0

0

0

0

0

0

0

0

0

0

0

0

0

0

0

0

0

0

0

0

0

0

0

3784

2713

2713

13

13

13

2532

178

146

0

0

32

2354

221

1939

188

6

168

4

4

1

0

0

0

1

0

0

0

0

1

1

0

8

8

0

0

0

144

0

143

1

1

0

1

0

9

9

0

0

0

0

0

7

2

2

0

0

0

0

2

2

0

0

0

0

0

0

0

0

0

0

0

0

2

0

0

0

0

0

0

0

0

0

0

0

0

1

1

1

1

1

1

0

0

0

0

0

0

0

0

0

0

0

0

0

0

0

0

0

0

0

0

0

0

0

3

0

0

0

0

0

0

0

0

0

0

0

0

0

0

0

0

0

0

0

0

0

0

0

0

0

0

3

0

0

0

0

3

3

136

135

0

0

0

2

2

0

0

0

0

0

2

0

0

0

0

0

0

0

0

0

0

0

0

0

0

0

0

133

0

0

0

0

133

133

0

0

0

0

1

1

1

1

0

0

0

0

0

0

0

0

0

926

0

0

0

0

0

0

0

0

0

0

0

0

0

0

0

0

0

0

0

0

0

0

0

0

0

0

0

0

0

0

0

0

0

0

0

0

0

0

0

0

0

0

0

0

0

0

0

0

0

0

0

0

0

0

0

0

0

0

0

0

0

0

9

8

8

0

8

0

0

0

0

0

0

0

0

0

0

1

1

0

1

0

0

0

0

0

0

551

551

459

0

459

78

0

14

1

2

0

11

22

28

14

14

0

0

0

0

0

0

0

0

0

0

0

0

0

0

0

0

0

0

0

0

0

0

0

0

0

0

0

0

0

0

0

0

0

0

0

0

0

0

0

0

0

0

0

0

97

97

1

1

0

0

96

0

5

2

0

5

38

0

0

0

10

5

0

31

0

0

0

0

0

0

0

1

1

0

0

1

0

0

0

1

0

0

0

153

151

0

0

0

0

63

63

12

12

6

6

0

0

0

0

0

0

70

0

70

0

0

0

2

1

1

0

0

0

0

1

0

0

0

0

0

0

0

0

0

0

0

0

0

0

0

0

0

0

0

1

0

0

0

0

0

0

0

0

0

0

7

7

0

0

0

0

7

4

2

0

1

0

0

0

0

0

0

0

0

0

0

0

0

0

0

0

0

0

0

0

0

108

29

0

0

25

25

3

0

3

0

0

0

0

0

1

0

0

1

0

0

0

0

0

0

0

0

1

0

0

0

0

0

0

0

0

0

0

0

0

1

1

0

0

0

0

0

1

1

1

0

0

0

0

76

45

45

0

0

0

0

0

0

0

0

0

24

24

0

0

0

0

0

0

0

0

0

0

3

3

3

0

0

3

1

1

0

0

0

0

0

0

0

0

0

1

1

1

0

0

2

2

2

2

0

2

0

0

1193

1193

1193

1193

0

0

1193

0

0

0

83

0

215

21

219

0

236

254

0

0

0

0

0

0

0

0

165

0

0

0

0

0

0

0

0

0

0

0

0

0

0

0

0

0

68

68

68

68

68

68

10106

10106

10106

9900

1

1

0

0

0

0

9899

8765

1134

206

206

26

28

40

10

14

21

18

49

0

3805

3805

3805

0

0

0

0

0

0

0

3805

3805

180

3616

9

0

0

0

0

0

0

0

0

0

1

0

0

0

0

0

0

0

0

0

0

0

0

0

0

0

1

1

1

1

1

0

0

0

0

0

0

0

0

0

0

0

0

0

0

0

3

1

1

1

1

1

0

0

0

0

0

0

0

0

0

0

0

0

0

0

0

0

0

0

0

0

2

0

0

0

0

0

0

0

0

0

0

0

0

0

0

2

2

2

2

0

0

0

0

0

0

0

0

0

0

0

0

0

0

0

0

0

0

0

0

0

0

0

0

0

0

0

0

0

0

0

0

0

0

0

0

0

0

0

0

0

0

0

0

0

0

0

0

0

0

0

0

0

0

0

0

0

0

0

0

0

0

645

0

0

0

0

0

0

0

0

0

0

0

0

0

0

0

0

0

0

0

0

0

0

0

0

0

0

0

0

0

0

0

0

0

0

0

0

0

0

0

0

0

0

0

0

6

6

6

6

6

0

0

0

0

0

0

0

0

0

0

0

0

0

0

0

0

0

0

0

639

639

0

0

0

0

0

0

239

239

239

0

0

0

0

0

0

0

0

400

54

54

0

0

0

33

33

0

0

258

258

55

55

0

0

0

0

0

0

0

0

0

0

0

0

0

0

0

0

0

0

0

0

0

424

424

424

424

0

0

379

140

174

65

45

45

0

0

62

62

0

0

0

0

0

0

0

0

0

0

0

62

62

62

62

0

0

0

0

0

0

0

0

0

0

0

0

0

0

0

0

0

0

0

0

0

0

0

0

0

0

0

0

0

0

0

0

0

0

0

0

0

0

0

0

0

0

0

0

0

0

0

0

0

0

0

0

0

0

0

0

0

0

0

0

0

15697

8168

1

1

1

0

1

0

0

0

0

0

0

0

0

0

77

77

70

70

7

7

308

0

0

0

0

0

0

308

0

0

308

308

13

13

13

0

13

0

2517

2291

9

9

1

1

266

252

2

12

0

148

41

107

0

0

0

53

52

1

0

0

0

4

2

2

1

1

0

0

2

2

0

0

6

6

1

0

1

0

18

0

0

18

0

0

79

0

6

1

0

5

0

0

0

0

67

0

0

0

0

0

0

0

0

0

0

10

10

41

41

44

0

43

0

1

57

1

49

7

130

4

4

0

111

0

11

22

2

0

0

2

16

0

0

2

0

7

7

22

0

0

0

22

0

158

158

22

22

29

29

28

23

5

1

1

0

0

0

74

74

5

5

0

0

59

0

11

48

0

0

0

0

0

40

40

6

6

0

0

0

0

0

0

1

1

172

9

163

1

1

0

539

0

298

0

52

0

153

3

0

0

0

0

33

235

165

70

0

0

0

226

226

0

226

4339

867

75

75

98

0

9

89

0

0

0

22

22

334

293

41

261

261

77

71

0

0

6

0

0

0

0

0

0

2705

1

1

0

0

1473

1473

36

36

1173

1173

0

0

22

22

0

767

0

0

0

0

0

0

0

0

0

767

767

0

0

4

4

0

0

0

0

4

3

1

0

0

0

0

0

0

0

12

0

0

0

9

9

9

0

0

0

0

0

0

3

3

3

0

0

0

0

0

0

0

0

0

859

644

0

0

0

1

1

34

34

0

0

0

4

4

0

0

2

0

2

0

0

0

0

0

0

0

0

0

0

0

206

3

0

1

202

0

3

1

0

0

0

0

0

2

0

0

0

0

0

55

55

91

0

91

248

0

1

0

247

25

25

25

0

0

0

28

0

0

0

28

28

0

0

0

0

0

0

0

0

0

0

0

0

0

0

0

0

162

0

0

0

0

0

0

0

2

2

0

0

1

1

0

0

0

0

0

0

0

0

0

0

158

158

0

0

0

1

0

1

0

0

0

38

1

1

1

0

0

0

15

15

15

22

22

22

0

0

0

4785

0

0

0

0

226

94

0

0

0

0

1

1

0

0

0

0

0

0

2

1

1

91

91

0

0

0

0

0

0

132

0

0

0

0

0

0

0

132

0

0

1

0

0

7

0

2

0

2

0

0

2

0

0

101

17

0

0

0

0

0

0

0

0

0

0

0

0

0

1011

611

0

0

0

0

94

94

6

2

0

1

0

3

0

504

504

2

2

0

0

0

5

5

400

21

21

40

40

0

0

0

0

0

0

16

16

0

0

0

122

122

1

1

10

0

10

12

12

3

3

2

2

39

39

0

0

100

100

34

34

0

0

0

0

102

15

8

8

0

0

7

2

5

87

87

87

0

0

0

0

0

0

0

1

1

0

0

0

0

1

0

0

0

0

0

0

0

0

0

0

0

0

0

0

0

0

0

0

0

1

0

0

0

0

0

0

0

0

0

0

0

0

0

0

4

0

0

0

1

1

1

3

3

3

0

0

0

1

1

1

1

0

3168

6

6

5

1

10

0

0

0

0

7

7

3

3

0

0

2319

2201

0

6

4

0

758

0

66

14

1353

0

0

0

0

118

8

110

6

6

1

5

825

1

1

1

1

13

8

0

5

11

0

8

3

134

0

103

2

0

0

29

9

9

339

14

1

319

5

0

0

20

20

283

238

12

33

7

5

2

4

4

3

0

3

2

2

2

0

0

0

0

0

0

0

258

258

258

0

62

45

0

151

0

14

14

0

0

1

1

13

13

0

0

0

0

0

0

0

0

0

0

0

0

0

0

0

2744

0

0

0

0

0

0

2744

0

0

0

954

0

0

0

0

0

0

419

1

418

535

109

93

60

15

0

79

24

22

133

1790

59

52

7

704

704

988

4

340

552

92

39

35

4

0

0

0

0

0

0

0

0

4

0

0

0

0

0

0

0

0

0

0

0

0

0

0

0

0

0

0

0

0

0

0

0

0

0

0

0

0

0

0

0

0

0

0

0

0

0

0

0

0

4

0

0

0

0

4

4

0

0

0

0

0

0

4

4

0

0

0

0

0

0

0

0

0

0

0

0

0

0

0

0

0

0

0

0

0

0

0

0

0

0

0

0

0

0

11

11

11

11

11

0

11

0

0

0

0

0

0

0

0

0

0

0

0

0

0

0

0

0

0

0

0

0

0

0

0

0

0

0

0

0

0

0

0

0

0

4

4

4

4

4

4

4

16

7

7

7

0

0

0

7

7

7

0

0

0

0

0

0

0

0

7

7

7

7

7

7

2

2

2

2

2

2
